# The Hospital Nursing Supplement

**Published:** 1894-08-11

**Authors:** 


"The Hospital" Nursing Mirror.

Being the Extra Nursing Supplement of "The Hospital" Newspaper.

[Contributions for this Supplement should be addressed to the Editor, The Hospital, 428, Strand, London, W.C., and should have the word "Nursing" plainly written in left-hand top corner of the envelope.]

### News from the Nursing World.

### THE NURSES' "AT HOMES."

The monthly "At Homes" for members of the Nurses' Co-operation, 8, New Cavendish Street, are likely to prove very pleasant and enjoyable functions. A daintily served tea is an acceptable accompaniment of these informal gatherings, and the nurses thoroughly appreciate the opportunity offered for meeting with old and for making new friends. The first Friday in each month is the date fixed for the nurses' "At Homes."

### A LESSON TO ALL.

Several lessons should be learnt by nurses reading the account of the recent death of a patient at Guy's Hospital. The man was shown at the inquest to have had an enlarged heart and chronic kidney disease, but his death was probably accelerated, it was stated, by an overdose of chloral. The doctor ordered a drachm and a half of the drug, and the nurse gave an ounce and a half instead, having mistaken the symbol on the prescription paper. Certainly the signs and abbreviations in general use are not always as distinctly written as they should be, but we are strongly of opinion that some knowledge of the ordinary doses of drugs in common use should be learnt by nurses before they are placed in charge of wards. Were the fact clearly impressed on every probationer's mind that *two* drachms of chloral is the maximum dose according to the British Pharmacopœia, no mere misreading of a sign would induce any nurse to administer three times that quantity. Thoughtful study, such as we have suggested, would be of practical use to most nurses, especially such accurate knowledge of poisons and their antidotes as would naturally be included in this relationship.

### DISTRIBUTION OF DIETS.

"Now, mate, wake up! it's dinner-time," says the cheerful patient in the accident ward. "All right," rejoins his neighbour; "I'm not likely to be asleep at feeding-time. Why, it's something to look forward to when you're in bed, ain't it?" Of course, dinner is made the event of the day by others besides hospital patients, and with less excuse for doing so. The latter have to take what is given them, and whether they eat it or not is a very important matter to a class of sick people who are not overfed at the best of times. Far too little attention is paid to the method of serving and placing food before patients in many institutions. In one, for instance, the tea is distributed by the ward-maids, the bread and butter cut by these worthy women being naturally of a most unappetising nature. At another institution, all the head nurses go to their own dinners whilst the newest probationers "distribute the diets," and in this case the supply of knives and forks, salt and bread is often somewhat eccentric. To see everything conveniently placed for the invalid before, not after, his hot dinner reaches him requires the experienced eye of a qualified nurse. A clean tray-cloth on the bed-table will reconcile many a fanciful woman to the meal for which she has but small appetite, and similar niceties of service have equally good results in tempting her to eat. The distribution of diets in a well-managed hospital is admirably organised, every member of the nursing staff taking a pride in helping to make a success of "the chief event of the day."

### THE MANAGEMENT OF MEASLES.

"You'll excuse the place being a bit untidy, ma'am; it's more than I can do to keep it straight when all the children's at home." "I'm sure you have nothing to apologise for, Mrs. Brown. Your cottage is always a picture," responded the Squire's wife pleasantly; "but I didn't know the holidays had begun." "No more they 'ave," retorted Mrs. Brown, rather sharply. "It's all that inspector chap's doings. He says none of my children's to go to school 'cos Mary and Martha's got the measles. 'Taint as if I wasn't a careful woman. Why, I've kep' them two separate most partickler." "How do you manage with them at night, Mrs. Brown?" "Oh, I just puts the other three to sleep in the foot of Mary and Martha's bed. I should never dream of letting 'em mix, and so I told the inspector; but he actually said that I'd no business to put them in the same bed. He just talked as big as if I didn't know as much about infection as he did!"

### "TRAINED NURSES" FOR SHROPSHIRE.

The President of the Ladies' Association of the Shropshire County Council read a paper on the training of nurses at a recent meeting in Shrewsbury Town Hall. It was reported in the local press that she showed "how the Technical Instruction Committee carried on its work in this direction, preferring women who had been good domestic servants, or widows who were already carrying on the work, and sending them for training for three or six months in maternity and general sick nursing." Surely neither the committee quoted nor the Ladies' Association can seriously expect to evolve competent nurses by means of such inadequate training. A careful perusal of the articles on district nursing which have appeared in The Hospital this year should convince the ladies of Shropshire of the injustice of providing the poor with less skilled attendants than are considered needful for the rich. Our beloved Queen sets an example which might well be studied and followed, in providing that only women fully trained in district as well as general nursing should be authorised by The Institute to undertake the charge of the sick poor, whose lives are surely as precious as those of their richer brethren.

### A SENSIBLE ENTERTAINMENT.

A day on the river, an afternoon in somebody's grounds, tickets for a concert, a long country drive, are amongst the pleasant changes which come occasionally to the hospital nurse, and those possessing the power seem increasingly willing to provide such treats for appreciative workers. But very few outings come within the reach of the workhouse nurse; she is still apt to be overlooked, just as the workhouses themselves have been hitherto passed over. However, one party of infirmary nurses had a charming outing secured to them recently, directly after their patients' early dinner and their own meals were over, as many as could be spared started off for a beautiful park, where everything was arranged to provide pleasure without fatigue for them. In a charming drawing-room—itself an education in modern art—afternoon tea was presently served. It was, of course, most acceptable, but the guests privately wished it had been a more substantial, if less elegant, meal, their own midday repast having been a hurried one. The time passed quickly in the lovely garden, and before the hour of departure the guests were ushered into the oak-panelled dining-room, and found a handsome early supper awaited them. Refreshed in mind and body, the nurses finally departed with grateful thoughts of the courteous lady by whom workhouse nurses are happily never "overlooked."

### WISBECH.

The Mayor of Wisbech has been interesting himself in the provision of a duly qualified nurse for the sick poor of that town. A management committee has been elected, and £100 per annum promised for the next three years. The scheme seems to have been well thought out and organised, and is therefore likely to work well.

### BARRY DOCK.

There is to be a sale of work at Barry Dock at the end of October in aid of the Nursing Association. Gifts are solicited, and few things will be too small to find a welcome, so long as they are useful and marketable. The work which is annually accomplished in the Barry and Cadoxton districts being of incalculable value, it is to be hoped that many friends will show their appreciation of the nurses' efforts by sending liberal contributions to The Home, 36, Kingsland Crescent, Barry Dock, for this autumn sale.

### LLANRHOS.

The Birmingham Hospital Saturday Convalescent Home is situated at Llanrhos, near Llandudno, and is for the exclusive use of men who reside or are employed within a radius of five miles from the Town Hall. Those recovering from illness and requiring change of air are eligible, but the committee reserves to itself the right to reject any application. During their stay at the home the patients are entirely under the control of the matron; in the garden they obey the orders of the gardener; in the farmyard they are subject to the steward. The rules are plainly printed, so that everyone knows beforehand exactly how many must be complied with.

### QUALIFIED AT BOYLE.

A novel application of a "Three months' course" is proposed by the Guardians at Boyle, who are anxious to install an untrained nurse in their hospital. After some discussion at their last meeting, it is reported by the local press that the chairman made the following minute: "The Board of Guardians are of opinion that Mrs. Kelly, who has already had a training at St. Patrick Dunn's as a midwife would, if allowed to remain on probation for three months, as has been done in Sligo, be likely to become thoroughly qualified. . . ." Their medical officer had previously presented a report, prepared by request in consequence of the questions asked by the Local Government Board. This report states that one thousand five hundred patients have passed through the medical and surgical wards in the last four years. Many important operations have had to be performed. "Those cases, both medical and surgical, in order to be properly looked after, require the care and attention of a nurse skilled in medical and surgical nursing. Mrs. Kelly, though a good midwife, knows nothing of such nursing." It is to be hoped that the Local Government Board will convince the Guardians that the three months' "probation," which they propose to give Mrs. Kelly as nurse of the hospital, means experience gained at the expense of the unfortunate patients over whom they wish to put her in charge, and that even by such dearly-bought "probation" she certainly cannot "become thoroughly qualified" in any branch of nursing besides that in which she is said to be already efficient. "Amputations at the hip," and other serious cases, are not likely to fare very well under such conditions as those proposed.

### BRISBANE.

The Brisbane Hospital has been for a long time past appealing for funds to build proper accommodation for its nursing staff, now about forty in number, besides wardsmen. The hospital buildings have received considerable additions since their erection on the present site in 1869, the special fever ward having been added in 1880. Brisbane Hospital stands in a particularly open position, and the wards are convenient and pleasant, and it is to be hoped that support will soon be forthcoming to secure a nursing home worthy of the institution. There is now accommodation for 230 patients, and the usefulness of the hospital is greatly augmented by its possessing a convalescent home, where the patients get the change and rest which aid so materially in their complete restoration to health.

### "INFANTS" AS NURSES.

"At what age can I begin to be trained in a hospital?" is such an oft-heard question that "Not under twenty-four or twenty-five" is answered instinctively. But this limitation is evidently not in force in all our colonies. Miss Blennerhassett's very interesting letter which appears in the present issue of The Hospital shows eighteen or twenty to be the usual age at the Kimberley Hospital. Therefore, the course of training at that institution can be apparently completed by girls who are legally infants. However admirable her organising ability, the youth of Sister Henrietta's probationers and their uniform are not in accordance with modern notions. We hardly think that even in well-managed colonial hospitals stuff frocks and girl nurses can be looked upon with general approbation.

### GUIDES TO KNOWLEDGE.

"I object to district nurses being ladies," remarked one of the local committee, "although no doubt they do their work well; but then one doesn't like ordering them about." But it seems not improbable that this has something to do with the preference in certain districts for the class of women who know less than her social superiors. The latter, who take it in turns to "look after" the nurse, are able, by virtue of their natural intelligence and a handbook or two, to give her hints, and yet being themselves untrained they are quite innocent of the terrible danger to which the sick are exposed when left to the mercies of unskilled persons, whose ignorant practices are protected and countenanced by bestowing the misnomer of "nurse" upon her. The thoroughly trained district nurse, who neither requires nor desires "ordering about," is a better friend to the cottager, and is always on good terms with a judicious committee.

### On General Nursing.

By Rowland Humphreys, M.R.C.S., L.R.C.P.Lond.

### XXIV.—TYPHOID FEVER—(*Continued*).

Meat before administration should be pounded or scraped or reduced to a pulpy consistence, or it may be made into beef powder or into a mould with the aid of gelatine. It may be predigested with the aid either of pancreatic fluid or of pepsin and hydrochloric acid. An excellent and nutritive beef-tea may be made by cutting a pound of lean meat, beef or mutton, into dice, and standing it in a cool place in a jar containing a pint of water for two hours, and then adding ten to twenty drops of diluted hydrochloic acid of the Pharmacopœia. The acid has the power of dissolving and digesting the albumen of the meat and is also of use as an antiseptic, as it is found that the bacilli do not develop in acid liquids. It must always be remembered by those who have to undertake the feeding of sick people that boiled beef-tea is useless from a nourishing point of view, as the nutritive material, the albumen, is thrown down by so doing. If the material thus precipitated be rubbed down and then stirred up in the broth, the liquid is very nourishing, otherwise it is purely a stimulant. The acid - made beef - tea may be boiled, however, provided this is done in a glazed vessel. If the acid taste be objected to, a very good beef tea may be made in a similar manner without acid, but cannot be boiled. Scraped raw meat is also very good, given in teaspoonsful. The raw extracts may be mixed with coffee, or Liebig, or may be disguised in any other suitable manner. It is necessary to caution those who have the making of raw meat preparations that they must be passed through a fine hair sieve, for fear of the presence of tape-worm eggs. The author, a little while since, had a case of tænia in a patient who had taken raw beef juice for some time as an article of food.

As already stated the patient should be induced to take plenty of liquid; barley water, distilled water in one of its aërated forms, or even injections of water into the rectum may be employed to assist this end. The action of the kidneys should be as carefully noted as that of the skin. Peptonised gruel, made with cornflower is also good; and although the patient may have little care for, or have little power of tasting food, still constant little changes are very agreeable, and ingenuity may be exercised to this end. The food should, especially, be pleasing to the eye, and nicely served, never bringing the same thing twice running, and only enough on each occasion to satisfy the immediate needs of the patient. It is always well to allow two hours, at the least, between each meal, in order to allow the stomach to empty itself and get a little rest, but the length of time beyond this which is allowed to elapse be tween one meal and the next depends on the amount of food which is being taken.

It has been necessary to enter thus fully into the feeding question as the treatment of this disease resolves itself, for the most part, into the administration of food, and of stimulants, reduction of fever, and treatment of complications.

In the first week, the principal thing to be guarded against is pneumonia and errors in diet, whether through taking wrong food, or too much food. Frequently a little hæmorrhage from the intestine or from the nose takes place, but it is of no consequence during this] week or the next one from a prognosis point of view.

There is usually more or less headache, and at times it is so continuous that the case has been mistaken for one of tubercular meningitis, a disease very like the one under discussion in respect to its earlier signs.

The fever keeps up during the second week, varying only a degree or two night and morning. It is usually highest at about five p.m., lowest in the early morning. The doctor often directs that a full dose of the antipyretic in use is to be given at about midnight, so as to prolong the morning fall of temperature. During this period the appetite is better and the patient more restful.

During the second week the doubt which is always present in one's mind as to the correctness of the diagnosis, is dispelled. The characteristic rash, if it is going to appear at all, shows itself. It may come on almost any part of the body but is most often seen on the abdomen, and back. It consists of small, oval, slightly raised spots, of a more or less pale rose colour, and if the skin on each side of it be streched by the separated fingers the spot disappears. Sometimes it comes out very thickly, at other times only a few spots appear; in about one quarter of the cases no spots at all appear, so that one gets in the habit of looking upon the spots as a confirmatory sign when present, but never expecting to find them.

The expression of the face and the appearance of the tongue are very characteristic. The apathetic, yet hectic appearance, the furred brownish tongue cleaning at tip and edges, all point in one directon. The diarrhœa commonly increases in frequency and in the amount passed at each evacuation, and, if antiseptics have not been administered internally, the fœtor from it is very offensive. The stools are pale yellow and fluid, and, if tested with litmus paper, are alkaline. They consist of the contents of the small intestine which have been hurried through the colon.

The spleen dulness is now well marked, the urine more or less scanty and high coloured, and there is a tendency to slight delirium.

During the first and second weeks an occasional aperient, such as "White Mixture" or a dose of calomel followed by a seidlitz powder in the morning may be required, and indeed is usually advisable, but during the third week if an aperient were to be administered there would be considerable danger of hæmorrhage from the ulcerated surfaces of the intestinal glands and neighbouring parts. Hæmorrhage in the third week is nearly always fatal; it rarely ceases until death or fainting occurs. In such a complication, ice and opium are the mainstays. The ice may be applied to the abdomen in small lumps between flannel, and lead acetate or other astringent administered, or ergotine given subcutaneously. The symptoms of hæmorrhage are loss of temperature, perhaps two degrees, blanching of face and mucous membranes, improvement in cerebral symptoms, and dulness over the colon, showing the presence of fluid (blood) in it. The blood when it passes may either be bright in colour or more or less tarry.

Another great danger in typhoid is peritonitis, either from perforation of the intestine by one of the ulcers, or from extension of the inflammation from the base of an ulcer. It is betokened by distension of the abdomen with flatus, by persistent vomiting, and by tenderness of the abdomen, and there will be some alteration in the regularity of the temperature chart, either a rise or a fall in temperature resulting.

Perforation is another great danger. It often follows hæmorrhage, and is usually fatal, unless the process of ulceration has been slow enough to allow of adhesions being formed between the intestines at the point where perforation threatens. Deep ulceration is said to be indicated by great tremor of the fingers or of the tongue, but the tremor must be persistent, and not from nervous excitement or other transitory cause. The author has often seen a very tremulous tongue without perforation following.

### Structure and Care of the Teeth.

### I.—DEVELOPMENT.

In considering the bony structure of the body we must not omit the teeth. The full complement of teeth in the adult, that is, the "permanent" teeth, amount to 32 in number—16 in each jaw. Many scientific men say that 44 and not 32 is the typical number, since animals belonging to the same class of mammals as man have 44, and therefore they say that certain teeth in man have been suppressed and never make their appearance. A knowledge of this diminution of teeth in man is interesting, as it explains to some extent the occurrence of what is sometimes set down as a third dentition, as well as the appearance of extra or supernumerary teeth in the mouth, the additional numbers in both cases merely showing a tendency to return to the normal or typical number, which had been departed from.

These 32 teeth are divided into four different kinds, and each division is held to present a certain typical shape or character, to which all teeth in the same divisions throughout the animal kingdom are referred. These divisions are called incisors, canine, bicuspid, and molar teeth.

As in the typical number of the teeth, so also in their typical form, great deviations occur throughout the animal world. These, however, must not be regarded as deformities, or as a departure from the usual pattern, but simply as due to modifications through necessity. This is another illustration of the fact that organs which are not needed tend to be suppressed, whilst those most used are retained and improved in their development.

The 32 permanent teeth in man are preceded by a less numerous set called the "milk" teeth, which fall out; this holds in the class of mammals, and then there are two generations of teeth, the temporary or milk teeth, which are destined to be shed, and the permanent or adult set, which succeeds them. These 32 permanent teeth in man are arranged as follows: In front, above and below, are four cutting teeth, flat or chisel-shaped at the edges, called "incisors"; touching these on each side is a conical, spear-shaped, piercing tooth, called the "canine" or "eye-tooth," behind this on each side, above and below, two small double-pointed teeth, called the "bicuspids" or small molars; behind again on each side, above and below, the large crushing or grinding teeth, the true "molars," making twelve in all. These true molars appear only once in a life-time, and only in the permanent set, and are not represented in the milk set, and the latter are only 20 in number instead of 32.

The typical form of each kind of tooth depends on the fang, or root, and on the crown of the tooth; thus the incisors and canine teeth have each only one fang, the bicuspids in the upper jaw usually have two, whilst those in the lower have only one fang. In the upper molars the usual number of fangs is two, and in the lower jaw three fangs each. This fact, of course, is of most use to the dentist who extracts teeth, though it may also be of interest to possible victims.

Man differs from all other mammals in the arrangement of his teeth. In man they are all uniform in length, being, as a rule, practically all on the same level at the crown, and every tooth being close to its neighbours. In lower animals, on the other hand, the series of teeth is interrupted by gaps, generally occurring near the eye-teeth; also the teeth are of various lengths, the eye teeth especially being much longer than their neighbours, and over-topping them considerably. The existence of these gaps in lower animals possibly accounts for their not suffering so much as man does from over-crowding, and the extra room so afforded may explain why the lower animals seem to suffer so much less during dentition than man does, in whom the jaw is barely sufficient to contain the mass of teeth in all stages of growth with which it is at one time encumbered.

The healthy growth of the jaw itself is, of course, an important element in the regularity of the permanent teeth by affording the necessary space, and its full and even large development generally shows bodily health, strength, and mental vigour and force of character. However, we are told that the tendency is for jaws to become smaller and less developed because they have less work owing to the improved preparation of food.

Let us consider shortly the development and structure of the teeth; how they first make their appearance, and whence they come, and how they grow.

At an early period in intra-uterine life the mucous membrane of the jaw becomes specially active at the joints where the future tooth is to appear. The development begins by this mucous membrane sending downwards certain finger-like processes, each of which will represent a tooth. The point or extremity of each process expands after a time till, when seen in certain directions, they look somewhat like a wine glass with a narrow stalk, the globular part lying deepest. Into the goblet-shaped end of the process a small projection of the subjacent tissue grows, and here is deposited the germ or beginning of the future tooth. The stalk portion of the goblet now disappears, and the goblet itself develops the enamel of the tooth, and the germ goes on increasing until it forms the substance known as "dentine," which forms the inner wall of the tooth itself, leaving a cavity in the centre. A part of the remains of this "germ" or "papilla" changes less than the rest, and forms the central pulp or "nerve" of the tooth which occupies the centre. Also an outside covering for the root is produced which closely resembles ordinary bone and is called "cement." As soon as the crown of the tooth is complete, and long before the root is perfect, the process takes place of "cutting" the tooth. This comes about, not by the tooth forcing a way for itself through the gum and other tissues, but by those tissues making a way through which the tooth appears.

Such is the process in infant life called "teething." This ought not to be regarded as a disease, but as a perfectly natural healthy process. Many persons, however, when any illness, no matter what, occurs in an infant at the age of teething, think it is due to this cause, and are anxious to have the gums and the delicate tooth germs just described laid bare, sometimes before they are ready, believing that this will cure the trouble. This is done in utter ignorance of the beautiful and carefully-protected structures with which they would interfere, and of the wondrous provision made for effecting what they rashly wish to hasten.

### A Case for Assistance.

After thorough investigation this case has been proved to be one requiring and deserving help. Miss Bell and Miss Farrow, Home for Aged Mariners, Egremont, Cheshire, are kindly receiving subscriptions for a nurse who was trained at King's College Hospital, filled the post of matron at several hospitals, and is now through ill-health and misfortune reduced to poverty. She is endeavouring to free herself from debt and to stock a small shop at Cardiff, in which she hopes to earn her own living. Nurse A. H. has sent 2s. 6d., and Sister Josephine (an old Mildmay worker), 2s. 6d.; making a total of £4 5s. 6d.

### Wants and Workers.

Can anyone tell me of a country or seaside home which would take in a girl of six, who has had scarlet fever (a slight attack), for a change when she comes out of hospital?—*E. M. K.*

### Character Sketches from a Lunatic Asylum.

### THE PATIENT WITH A GENIUS FOR ORGANISATION.

WHAT asylum does not possess, and what asylum nurse does not know, to her sorrow, the patient who has a genius for organisation? Who knows the private affairs of everyone in the place, or professes to know them. Who can tell you how the institution ought to be managed, what improvements are required, and what is the best way to do every kind of work. Who is always threatening to report the nurses for neglect of duty, and, at the same time, by constant interference, makes the performance of duty as difficult as possible. Most troublesome patients are these to manage, the more so that they are generally intelligent and quick witted, and that very often the chief symptom of their insanity is that they must have a finger in every pie they can find.

Such a one I well remember, whose perseverance in well-doing was calculated to overcome any obstacle. And, indeed, she had obstacles to overcome which nature had placed in her way, for her one arm was withered and useless, and she was lame through one leg being shorter than the other. She made up for any physical deficiencies, however, by the abnormal activity of her brain and her tongue, neither of which ever took any rest except when she slept, and that was comparatively little. She pervaded the whole place, giving orders both to patients and nurses, without respect of persons, setting everyone to work and standing over them to see that the work was properly done. She knew something about everything, and if by any chance there was anything she did not know, she never ceased asking questions until she obtained the information she wanted.

No amount of persistent snubbing availed to suppress her. She was told to mind her own business, with brutal plainness, fifty times a day; but other people's business had an attraction for her that she was powerless to resist. If she saw two people in conversation, no matter if they were visitors, and perfect strangers to her, she had no compunction in standing close to them and listening to every word they said; and her memory was a perfect storehouse of private family histories and gossip of every description.

She spoke to the other patients with such an air of authority, that new comers were apt to think that she really held some responsible position. When they found that this was not the case they naturally resented her interference, and this led to endless quarrels. Indeed, there was no patient equal to her for upsetting everyone, and putting a perfectly quiet room into an uproar in about five minutes. If she could not leave well alone, still less could she leave ill alone (which, I may remark, it is often wise to do in a lunatic asylum), and it was hardly possible for anything to be done right unless she had done it, or at least supervised it. From the management of the institution to the arrangement of the flowers in the day-room, she had a word to say about everything, and where she could possibly make an alteration herself she was sure to make it.

Her bright keen eyes saw everything, and she was never at a loss for words. How it was that her tongue never tired I cannot tell; but even when reading to herself she always read aloud, and after she was in bed at night she would continue to talk for hours, till she was the despair of the dormitory and of the night nurse.

Yet I must not be unjust to her, for she had many good qualities, and, when all was said and done, almost everyone was fond of her. She was really warm-hearted and good-natured, and both willing and anxious to work, as far as she was able. It was, indeed, wonderful what she accomplished with her right hand alone, for the left hand was perfectly useless. She was much more industrious than many patients who had the use of both their hands. Though she could not do needlework, she could manage to work book markers and slippers in cross-stitch; and she would help in washing up, in dusting and tidying the room, and in many such little things, most cleverly. She could write a very fair letter, too, and would often write letters for other patients who were too lazy, or too uneducated, to do so; moreover, she not only wrote the letters, but suggested what they should say. This, no doubt, was one of the ways in which she made herself acquainted with people's private affairs.

If anyone was laid up, she was most kind and attentive. She would run to and fro, fetch and carry, tidy the room, fill the water jug, or anything that she was asked to do. She would also read aloud, or entertain the invalid with the choicest bits of scandal she could recollect, and she was able to sing, energetically, if not very melodiously.

Her deformity was a great drawback to her in two ways. In the first place, if she had been able to occupy herself more regularly—with needlework, for instance—she would very likely have been less restless and interfering. On the other hand, her helplessness made her more or less an anxiety to the nurses, because she could not be kept from constantly aggravating and annoying the other patients, and if one who was violent should turn upon her she was quite unable to defend herself. It showed how utterly without self-control she must be, that she could not prevent herself from interfering with and aggravating patients whom she knew to be violent and able to hurt her, and who perhaps had already hurt her more than once.

She was a great alarmist, and if anyone received a blow or a cut she ran about announcing that they were killed. However, those who were used to her never troubled about her startling statements, and she was often useful in calling the attention of the nurses to small accidents or in summoning help when it was required.

It seemed sad that she should pass her days in an asylum, yet when free from restraint she was an intolerable nuisance to her family and her neighbours. Her temper was roused by the smallest trifle, and, once roused, was quite beyond her control. At such times it was only her comparative weakness and helplessness that made her easier to manage than other patients with violent tempers.

Although a middle-aged woman, she was in many respects like a child, and was always treated as such. She became very abusive when put out or when rendered irritable by one of her numerous small ailments, of which she always made the most, and thought herself greatly aggrieved if they were not immediately attended to.

On the other hand, her outbursts of temper passed over as quickly as they began; her anger was easily turned away, and then she was so sorry for what she had said, and so anxious to make up for it in some way, that one always forgave her on the spot.

She could be a most amusing companion, and it was a pity that her uncontrolled temper and mischievous tongue spoiled the really amiable qualities which she undoubtedly possessed. As a very intelligent patient once observed to me, "it was like a cow giving you a good bucket of milk and then putting her foot in it"—a comparison so apt that no words of mine can possibly improve upon it.

### Where to Go.

THE class-room at the Trained Nurses' Club, 12, Buckingham Street, Strand, can be hired by the day or hour for lectures, classes, committee meetings, &c.

PREPARATION CLASSES for L.O.S. examination in October will be held on Mondays and Thursdays in September at half-past four at the above address.

### Cholera Precautions.

The Ladies' Sanitary Association, 22, Berners Street, London, issues the following instructions in good clear type on a large card designed for hanging on a wall. It is a matter of regret that neither the strength nor the kind of "disinfectant solution" advised for clothes should be given:—

1. As cholera is not, in the ordinary sense of the term, contagious, not being communicated in the same manner as small-pox or scarlet fever are—directly from person to person—those coming in contact with it or engaged in attendance upon patients, are not more liable than others to become attacked if they take the necessary precautions.

2. All states of alarm, fright, or apprehension should be allayed, as it is certain that these only favour the development of the cholera poison. Confidence should be encouraged, and that manner of living pursued which we know to be best for our health.

3. The dwelling house should be kept clean, thoroughly dry, and well ventilated. The drains and their traps, as well as closets, should be examined and kept in perfect working order. Dust bins should be frequently emptied and disinfected. No refuse heaps or decaying matter of any kind should be allowed to remain in or near the house. Water cisterns, reservoirs, or butts should be frequently inspected and cleansed. All connections of waste or sink pipes with drains should be interrupted.

4. As water is one of the chief agents by which choleraic infection is conveyed, all water for personal or domestic use shonld be carefully guarded from contamination of every kind. The water should be boiled, afterwards filtered, and used within twenty-four hours.

5. With regard to diet, the meats may consist of any sort of animal food, fresh and green vegetables, all thoroughly cooked; bread, well boiled potatoes, and plain farinaceous puddings. Fruits, if wholesome and ripe, should only be taken in moderation, and after being washed and wiped; the skins should not be eaten, they are safer cooked. Milk should be boiled before use. Alcoholic beverages should be avoided. It is desirable to avoid the following: Soups, tinned or preserved meats, raw or stale vegetables, unripe or over-ripe fruits, pastry, cheese, nuts, malt liquors, ginger beer, acid wines, coarse oatmeal, and everything which one has found by experience to disagree. As a rule, during the presence of cholera, avoid sloppy and liquid foods, and take at each meal some form of solid nourishment.

6. All provisions should be procured fresh, but if they have to be stored, great care should be taken to protect them from contamination by impure air or water. Cooking utensils should be scalded after use, and kept carefully clean.

7. Keep the bowels regular, so as to ensure going to the closet daily—every morning if possible. Avoid the use of strong aperients, especially the saline kinds, as salts, citrate of magnesia, &c. If an aperient should be required, take at bed-time a teaspoonful of Gregory's powder, or one or two teaspoonfuls of castor oil.

8. Avoid exposure to cold or wet weather without sufficient clothes or protection. Keep the feet dry and warm. A flannel belt worn round the waist is very useful and a safeguard against chills. Take moderate exercise, lead a regular, quiet life, avoiding fatigue, excitement, mental strains, and everything that irritates or exhausts the nervous system. Especially avoid flying to alcoholic stimulants to remove feelings of sinking or depression.

### The Management of an Attack of Cholera.

When looseness of the bowels sets in, send at once for medical assistance, and in the meantime get to bed, between blankets, with a hot bottle to the feet. Choose if possible a bright airy room. Keep quite warm, and if troubled with cramps or pains, apply hot applications to the abdomen.

If thirst be excessive, sip from time to time small quantities of iced water, acidulated with aromatic sulphuric acid, in the proportion of one teaspoonful of the acid to a pint of iced water. Medicines will be prescribed by the doctor, but in his absence, and if the symptoms are urgent, the following mixture may be procured and given every three hours:—

Tinct. Camp. Co. and Tinct. Chloroform Co., of each half a drachm in an ounce of peppermint water, for one dose.

The diet should consist of boiled milk thickened with rice flour or biscuit powder, also of tea made with boiling milk infused about five minutes, with toast or rusks soaked in it. Also of farinaceous puddings made half fluid, gruel, meat jelly, beef tea, mutton, chicken, or veal broth. A large cupful of one of these should be taken every three hours.

From the first appearance of looseness of the bowels the body should be kept very warm. All evacuations are highly poisonous, and should be received at once into a vessel containing about a wineglassful of a saturated solution of sulphate of iron (copperas). Soiled bed or other clothes should be at once disinfected and destroyed, or steeped in a disinfectant solution, and afterwards boiled. Blankets should also be steeped in a disinfectant solution, and rinsed through boiling water. Soiled mattresses should be disinfected or burnt. The nurse or those visiting the sick-room should wash their hands and faces in carbolised water before partaking of food, which should never be eaten in the sick-room. Disinfection and fumigation of the room and its contents, when disused, is always necessary.

### The Bedford Workhouse.

### RATEPAYERS' RIGHTS.

The "ratepayers' money" is often emphatically mentioned where guardians seek to justify small acts of parsimony, and the "ratepayers' rights" ought to obtain at least equal consideration. They do not seem to be acknowledged at Bedford, where the master, as asserted in Mr. Stanley Lane-Poole's letter in the *Bedford Times*, last week refused admittance to a lady who has been a regular weekly visitor for twelve months. The paupers have been permitted to receive from her tea, tobacco, and toys. She has been consulted about nursing matters. The master's sudden refusal to permit her further visits requires explanation, and so do several of the recent actions at Bedford Workhouse. Attention has been already called by the Press to the dismissal, at a month's notice, of a valuable nurse because she refused to sever her connection with the Workhouse Infirmary Nursing Association. The *Bedfordshire Mercury* says that a month's notice was given by nine guardians after about twenty-four others had left the room. Doubtless the latter will see fit to enquire why a matter of such importance was brought forward and settled at the fag end of the meeting, when the majority of the guardians and the representatives of the Press had departed. The slight put upon the Association is of comparatively small moment to so well-known a society, but the offence has been considerably aggravated by the clerk to the guardians writing, in answer to a request from the secretary for the reasons of the nurse's dismissal, "the guardians do not recognise the right of your Association to question them upon such matters."

### Appointments.

Dewsbury District General Infirmary.—Miss Sara Jacobs has been appointed Matron of this infirmary. She was trained at the London Hospital, and afterwards worked at St. Bartholomew's Hospital, Lincoln County Hospital, and National Hospital, London. Miss Jacobs has also lectured for the National Health Society. We wish her every success in her new post.

Borough of Portsmouth Association for Nursing the Sick Poor.—Miss Helen M. Gibbs has been made Superintendent of this association. She was trained at the Royal Infirmary, Edinburgh, and the National and Metropolitan Nursing Association, Bloomsbury, London. Miss Gibbs has been in charge of the Addlestone (Surrey) branch of the Windsor Association for Nursing the Sick Poor, and we congratulate her upon her present appointment.

Caterham Asylum for Imbeciles.—Mrs. Warren has been appointed Matron of this asylum. She has worked for the last five years at the Chelsea Infirmary, where she has filled, with marked ability, the posts of Staff Nurse, Night Superintendent, and Assistant Matron successively. Mrs. Warren enters on her new duties in October next, and we wish her every success.

Alnwick Infirmary.—Miss Pitchford has been appointed Nurse-Matron at this infirmary. She was trained at the Royal Infirmary, Preston, and afterwards held the post of Surgery and Theatre Nurse; assisted the matron in the housekeeping, gaining a thorough knowledge of hospital management. Miss Pitchford had charge of the male surgical and medical wards for two years, and has had experience in the female wards also. We congratulate Miss Pitchford on her appointment, and she takes with her the good wishes of those who have known her for nearly six years at Preston.

### Women's Work.

### WOMEN AND PHYSICAL EDUCATION.

Many and remarkable have been the changes that have come over our social life during the last twenty years, and amongst those of the greatest importance may be reckoned the increased attention paid to the physical training and education of women. We have discovered that women can advantageously practise physical exercises as well as men, and indeed, was it not Richard Jefferies who wrote, whether rightly or wrongly, that "A full-grown woman is physically stronger than a man. Her physique excels man's." We will not discuss that question here; it may be so, or it may not.

Physical training, then, began when women took to croquet, and from that went on to lawn tennis, cricket, swimming, cycling, golfing; in fact at the present moment there is hardly any athletic sport or exercise in which women do not take their full and active share.

And what has this done for women? In what way has it affected them? We need not go far to seek the answer. It has made them stronger and more able to endure, and with increased strength they have gained in the development of body and limb, and not only developed physically, but mentally also.

The wisdom and necessity for the physical training of women having become so universally recognised, the need has sprung up for competent qualified teachers; in fact, remarkable as it may seem, the demand is greater than the supply, an unusual state of affairs in women's work, and, in consequence, the teaching of gymnastics is one of the most promising fields into which a woman may direct her energies, for, instead of having only a past, it has a future, and a bright future, too. Many women are specially adapted for this work, and for them it must have many attractions.

There are many systems of teaching gymnastics, some of them very good. Perhaps the "Ling" system is one of the most successful. This system is taught at the Hampstead Physical Training College, Broadhurst Gardens, N.W., where the training is very thorough and practical.

The course, which is divided into two parts, extends over a period of two years. The theoretical course includes the studies of anatomy, animal physiology, animal mechanism, hygiene, and elementary pathology; the practical course embraces gymnastics (Ling system), fencing, swimming, dancing, wood carving, medical gymnastics, massage, and outdoor games. Moreover, work is guaranteed to pupils on their obtaining their certificates at the end of their two years' course, when examinations are held (South Kensington Advanced Science, and College examinations). It is very seldom that work and a salary of £100 a year can be guaranteed at the end of a course of training, but this, we are told, is the case in this branch of women's work. The system is taught in most important schools and colleges in England; it is adopted at Girton, Newnham, Cheltenham, Bedford, and others; also in many of the principal training colleges, such as the Maria Grey, Cambridge Training College, Whiteland's, and St. Katharine's. The cost of the training is fifteen guineas a term, these terms including admission to the swimming baths and the examination fee at the Science Department, South Kensington; with board and residence it is thirty-five guineas a term. The age of admission is eighteen, and a sound constitution, a well-developed body, a thorough education, a fair amount of intelligence, and an aptitude for the study of natural science are necessary for a student. Occasionally it happens that a girl is not fit at the end of her two years' course to be sent out to a position, it may be from youth, abnormal nervousness, or any other cause. Under these circumstances she has a third year of training without any additional expense, a certificate never being given until a pupil is really competent.

The British College of Physical Education, 92, Long Acre, W.C., works on somewhat different lines. The name is rather misleading, for it was really established for the purpose of forming a centre for all interested in physical education, the aim of the college being to get into touch with all those in any way connected with physical training, so that all might gain by the united experience of those foremost in this movement. It also establishes a standard of excellency, gives a diploma, and superintends the conduct of the examinations, which are held twice a year, in May and November. This examination is divided into two parts, theoretical and practical, and they may be taken, if desired, at different times. The practical examination includes personal performances on the horizontal bar, parallel bars, rope climbing, vaulting horse, inclined ladder, and jumping; and in addition to this, candidates have to teach a class before the examiners in drill, mass exercises, free movements in the use of dumb-bells, wands, and Indian clubs. By passing this examination and becoming a registered member of the college, a guarantee is offered to the public that a woman is qualified to give physical training on scientific lines, one of the principal reasons for the existence of this college being to ensure competency.

Those meditating upon the inevitable question, in these days, of how to earn a living, may see in this sphere of work an opening in which there is every chance of success, for it is obvious that it is sometimes wise not to direct our attention to professions and employments already overcrowded, but occasionally to inquire into the advantages of "Fresh fields and pastures new." A. A.

### Hints on Disinfecting Rooms.

In answer to our suggestion that we should be glad to hear of the methods employed by nurses on whom the responsibility of disinfecting a room may rest, we have received, amongst others, the following communications:—

### By a Queen's Nurse.

Everything which has been worn or used by a patient in illness should be left in the room, scattered on the floor or hung on chairs. Bedclothes, the bed, and the mattresses should be turned up. All letters should be burned, and books and work opened and spread about. All cupboards opened and cleared, and all windows shut and fastened, and every crack or crevice pasted over with strips of paper (except the door of exit); chimney well stuffed, and pasted over with paper to exclude all air. Then a footpan, with one or two inches of water, is placed in the centre of the room, with a good margin all round. An iron pan or trough should be put in the pan, and if the room be good sized—say 18 ft. by 20 ft. —three pounds of rock sulphur laid in the trough, and when all is prepared a few red-hot coals should be put on the sulphur, and the room immediately quitted, the door locked, and paper pasted outside over every crevice, and left for twenty-four hours. Next day the room should be thrown open for twelve hours, windows cleaned, woodwork scrubbed with Calvert's carbolic soap, ceilings and walls scraped (which is most important) and washed down with carbolic solution 1 in 80, and the room will be free of infection.

### By Another Nurse.

If left to disinfect a room I should proceed in this way. All articles of wearing apparel which have been used by, or in any way come in contact with, the patient I should hang on a line across the room. All crevices around windows I should carefully fill up. I should then put a sulphur candle in a large bowl and place it on the floor in the centre of the room and light the candle. Then go out, fasten and seal the door, and leave it for twenty-four hours. Then I should throw open door and windows, and after a few hours' airing the cleaning should begin. All furniture, floor, paint, &c., should be washed with Sanitas, Jeyes' Fluid, or carbolic lotion. Walls and ceiling should be limewashed.

By "F. W. J."

See that all windows and doors are not only closed but properly blocked up, also fireplace and all crevices. Then place an iron vessel in the centre of the room with sulphur (one pound to every 1,000 cubic feet of space). I prefer McDougall's prepared sulphur cakes. They are supplied in 1 lb. and 2 lb. cakes. All articles of clothing and bedding should be hung upon a line. Everything being ready the sulphur may be lighted. If the cakes are used they only require a match put to them, but if "lump" is used it can be lighted by placing a live coal on it. Close the door and paste paper all round and leave for twenty-four hours. Then open windows and doors for twenty-four hours to allow of free ventilation. This being done, all paper should be removed from the wall and burnt, and ceiling whitewashed and woodwork washed with soft soap or Hodgson and Simpson's carbolic soap.

By an Experienced Nurse.

Although the entire responsibility of disinfecting a room after an infectious disease has been notified in a private house can be left in the hands of the sanitary officer, yet it is obviously necessary for every nurse to understand thoroughly how the process can be best carried out. It is also most desirable to fumigate rooms where no notifiable disease has been present, and a nurse should always ask leave of the friends to thoroughly disinfect chambers where patients have died of cancer, phthisis, and many other complaints. It is very satisfactory to find nowadays that the old-fashioned and elaborate plan of using a shovel, tongs, basin, brick, &c., is no longer necessary to effect fumigation. A neat little tin is now supplied which contains about twenty ounces of sulphur dioxide (sulphurous acid gas) "condensed into a liquid by pressure, and is equal in effect to about double its weight of sulphur as ordinarily burnt for disinfection." Full directions for use are given on each tin, and the process of disinfection is completed in eight hours. Messrs. A. Boake, Roberts, and Co., Stratford, London, are the inventors of this simple and efficacious appliance.

### Notes and Queries.

### Queries.

(109) *Holland Institute.*—Will you kindly tell me at what date the nurses connected with this institution begin work in the autumn?—*Enquirer.*

(110) *District Nursing.*—I am told that some capital papers on this subject have been issued from time to time in The Hospital, and I shall be grateful for information as to the approximate dates of their appearance.—*A New Reader.*

(111) *Convalescent Homes.*—Will you kindly furnish me with names of some convalescent homes for children in Ventnor or Bournemouth?—*H. N.*

(112) *Infection.*—Is there any infection about "peeling" in scarlatina at the end of six weeks?—*Nurse Ell.*

(113) *Fumigation.*—Kindly tell me whether the inspector is responsible for the disinfection of private houses.—*Nurse.*

(114) *District Nursing.*—Can you tell me of a book on this subject?—*Spring Time.*

(115) *Army Nursing.*—Please tell me to whom I should apply for an appointment as an army nurse.—*Nurse Emma.*

### Answers.

(109) *Holland Institute (Enquirer).*—The directress of this institute advertises in The Hospital towards the end of September for nurses to fill such vacancies as occur. This is early enough for applications to be made, as engagements are usually entered upon in December or January.

(110) *District Nursing (A New Reader).*—In The Hospital of January 6th, 1894, appeared the first of an excellent series of eight articles on District Nursing. In the issue of May 12th, 1894, there was a valuable article on "The Advantages and Privileges of a Trained District Nurse."

(111) *Convalescent Homes (H. N.)*—You will find the information you want in "Burdett's Hospital Annual," which is published by the Scientific Press, 428, Strand, London, W.C.

(112) *Infection (Nurse Ell).*—Certainly as long as ever "peeling" continues there is danger of the patient infecting other persons.

(113) *Fumigation (Nurse).*—The sanitary inspector is entirely responsible for the proper disinfection of a house where an infectious notifiable disease has occurred. If a nurse has been employed she will of course see that things are put in proper order before leaving the infected room.

(114) *District Nursing (Spring Time).*—See answer to query (110) above.

(115) *Army Nursing (Nurse Emma).*—If you have had three years' hospital training you should apply to the Director-General of the Army Medical Department, Victoria Street, Westminster.

### For Reading to the Sick.

### COMPASSION.

**Motto.**

Pity and need make all flesh kin.—*E. Arnold.*

Unless you are deliberately kind to every creature, you will often be cruel to many.—*Ruskin.*

**Verses.**

Teach me to live—my daily cross to bear,
Nor murmur tho' I bend beneath its load;
Only be with me; let me feel Thee near;
Thy smile sheds gladness on the darkest road.

Teach me to live and find my life in Thee,
Looking from earth and earthly things away.
Let me not falter, but untiringly
Press on, and gain new strength and power each day.

Teach me to live; with kindly words for all,
Wearing no cold, repulsive brow of gloom,
Waiting with cheerful patience—

**Reading.**

Bear ye one another's burdens, and so fulfil the law of Christ.—Gal. vi. 2.

Those who know much of the trials of invalidism from personal experience, will agree that special forms of temptation assail the sick room, so that it becomes to its occupants, as the wilderness was to our Lord, a very battle field!

We must not suppose, however, that the patient is the only sufferer, or the only one who has to confront the fiery darts of the Evil One. The nurses who watch by the sick beds and are in constant attendance on sick folk, have fully as much need to fall back on the promise of God's help as the sick ones themselves. It is not easy at all times to possess the soul in patience when shut up in a room with whimsical, exacting people, who seem to think by virtue of their affliction that they have a right to demand the lion's share of attention, and appear to think themselves the pivot on which the whole world turns It needs a great deal of grace to bear patiently with such; to be perfectly just to them in thought as well as deed . . . to be tender and pitiful and compassionate, willing to bear as far as possible their burdens, and to think about the Son of Man, who came not to be ministered unto but to minister. And it needs a double portion of grace to be willing to be taught to exercise the sweet graces of hopefulness, cheerfulness, and above all self-forgetfulness. . . . When our whole body is sick and faint, and our feet weary of running hither and thither . . . it will help us to remember that it was at the time of Christ's bodily weakness, through fasting, that the devil sought to gain an advantage over Him. Opportunities for triumphing over self are of continual recurrence to both nurse and patient in the sick room; it is in an especial manner "a road" in which "there is room to deny ourselves," and if patiently walked in shall indeed "lead us daily nearer God."—*Mary E. Kendrew.*

Touched by the love of Christ . . . compassion will gain for us again its true meaning. We shall minister to the weak and erring, not in condescending pity, but as enabled to share evils which are indeed our own.—*Westcott.*

Sympathy should especially be wrought out in us by sickness. No sick persons have truly understood the lesson that it was designed to teach them until they have learned this truth. We are very slow to learn true sympathy. . . . Sickness wrongly received increases selfishness to the highest degree. Sickness rightly received does by degrees cast out the "unclean spirit" whose "name is Legion."—*Sickness; its Trials and Blessings.*

And whether one member suffer, all the members suffer with it; or one member be honoured, all the members rejoice with it.—*St. Paul.*

☞ For The Muses' Looking-Glass, Everybody's Opinion, Book World for Women and Nurses, &c., see page ccix. et seq.

### The Muses' Looking Glass.

### ROUND THE LONDON HOSPITAL.

An infallible panacea for a fit of the blues we have found, from frequent experience, to be obtained from a quarter of an hour's intercourse with happy-hearted Chaucer. He strikes in his songs no strings which vibrate to the tune of "Is life worth living," either for oneself or one's neighbours. Yet he had his troubles, too, both inward and outward, which he bore like a man, and from which, as an earlier Wordsworth, he sought solace in the contemplation of the simple things in nature.

"When that the monethe of May
Is comen, and that I hear the foules synge,
And that the floures gynne for to sprynge,
Fairewel my boke, and my devocioun,
Now have I thanne suche a condicioun
That of alle the floures in the mede
Thanne love I most, this floures white and red,
Suche as men callen daysyes in the towne."

It requires an effort to overcome the depressing effect of the present "sordid dead level" conditions of existence witnessed in walks at the East of London, and a still stronger imaginative effort is demanded of one who would realise Whitechapel as it looked from Chaucer's windows when he lived over the gateway at Aldgate.

In 1374 a lease was granted to Geoffrey Chaucer of "the whole of the dwelling-house above the gate of Aldgate, with the rooms built over, and a certain cellar beneath the same gate, and the appurtenances thereof," on condition that he "will completely and sufficiently maintain and repair" the same on pain of being "ousted." The poet was not on any account to be allowed to let the house, and in time of defence the Mayor or his officials reserved the right to enter or deprive the tenant of the rooms at discretion.

The stately gateway stood across the High Street south of Aldgate Church; in 1215, the Barons, at war with King John, found entrance to London at this point so easy, that after using it they rebuilt the gate.

Chaucer was one of the last persons permitted to rent houses over gateways, for in 1386 the City enacted that they should not be let as private residences in future.

The poet, oppressed with his hopeless love affair, apparently loved the society of books and daisies better than that of humanity at this time, for the eagle in the "House of Fame" upbraids him that—

"Noght oonly fro ferre contree,
Ther no dydynge cometh to thee,
Not of thy verray neyghebors,
That duelle almoste at thy dors,
Thou herest neyther that nor this,
For when thy labour doon al ys.
* * * * *
Thou goost home to thy house anoon,
And also as dombe as any stom,
Thou sittest at another booke
Tyl fully dasewyd ys thy looke."

Queen Mary entered London by Aldgate, her sister Elizabeth receiving her with affectionate welcome, and an escort of 2,000 horse. The gate was taken down in 1606, and replaced by another more in keeping with the taste of the 17th century. The Lord Mayor's carver—an official with no sinecure—had his dwelling over Aldgate in the 18th century, but carver and gate were alike swept away from the High Street in 1761.

Did Chaucer ever dream of his lady in "the garden at the south side of Aldgate, called the Hermitage, which Roger Atte Wattre held" in 1325 "for a yearly payment of ten shillings?"

"A draft on Aldgate Pump" had an ugly sound to bill discounters in the 18th century, but the pump itself was an old institution, and is mentioned by Stowe—it was only closed in 1876—the well was found to be tainted, and the present drinking fountain erected in its stead.

Close by the pump a Prior of St. Katherine's or of Holy Trinity founded a church about 1110, and until about the year 1870 an early English crypt, probably part of a later addition to the church, remained under one of the houses near.

The great Priory of Holy Trinity without Aldgate was founded by Henry I.'s queen, the Saxon Matilda; the priors were Aldermen of the city, and the monastery was of great importance until Henry VIII. granted it to one Thomas Audley, from whose family it passed, by the marriage of an heiress, into the possession of Thomas Howard, whose title is commemorated in Duke's Place and Street.

Another very beautiful building in Chaucer's day was St. Katherine's by the Tower. This Royal hospital, college, and "free chapel" was originally founded by Matilda, the wife of King Stephen, as it has always been specially connected with the consorts of English monarchs. Giddy little Queen Eleanor of Provence did much for St. Katherine's after her husband's death, and good Queen Eleanor, to whose memory Edward I. erected the crosses, refounded it; while another virtuous lady, Phillipa of Hainault, further enlarged it. Katharine of Aragon founded a "Guild of St. Barbara" here, of which Cardinal Wolsey was an ornamental member; but the Reformation swept away St. Barbara, and Elizabeth, by a charter of 1556, provided for the maintenance at St. Katherine's of a master, three brethren, three sisters, and ten bedeswomen. The requirements of modern commercial life led to the removal of St. Katherine's in 1825 to Regent's Park, and that is, as we know, the present address of the superintendent of Queen Victoria's Jubilee Nurses.

About Charles II.'s reign "Whitechapel was a fair, spacious street for entrance to the city eastwards . . . . being the Essex Road, and well resorted unto . . . . it is the better inhabited and accommodated with good inns for the reception of travellers, and for horses, coaches, carts, and waggons." Defoe lived "midway between Aldgate Church and Whitechapel" during the great plague, and he used to watch the frightened richer inhabitants of the stricken city flocking out to the country by this route.

Poplar derives its name from the trees which loved its damp soil, and in 1720 there were "yet remaining in that part of the hamlet which bordereth upon Limehouse, many old bodies of large poplars standing, as testimonials of the truth of the etymology."

Limehouse is not connected with lime trees, but is called after the "Lymechostes," where, as early as August 17th, 1417, an inquest was held over the body of a "steersman or bodysman" of a ship, who met his death near the kilns.

Dickens' Smollet-like character Rogue Riderhood in "Our Mutual Friend" dwelt in Limehouse Hole.

The present church of St. Mary's, Whitechapel, has had two predecessors—the red brick structure put up in 1675, and a still earlier church of the decorated period which that replaced. Dr. Walton, the clergyman here in 1710, was "suspected to be a Jesuit," apparently because he had Dean Kennet, with whom he was at enmity, represented in an altar-piece as Judas, to the scandal of the diocese and the confusion of the painter.

But the strangest thing about this church is its name. In 1336 a "Cardinal Bishop of Alba, parson of Stebenhith," presented a clerk to the living of the "Church of the Blessed Mary called Matfellon, without Aldgate," therefore the explanation that the title Matfellon refers to the murder of a thieving Frenchman by the ladies of Whitechapel in 1428 is dated too late to be satisfactory.

### Everybody's Opinion.

[Correspondence on all subjects is invited, but we cannot in any way be responsible for the opinions expressed by our correspondents. No communications can be entertained if the name and address of the correspondent is not given, or unless one side of the paper only be written on.]

### THE MATRONS' COUNCIL.

"A Matron who Was Present" writes: In reply to Miss Benger's letter. When a matron objected to the name of "Matrons' Council" if sisters, &c., were to be admitted, "which was done" when the first bye-law was under discussion, she was asked to wait until the third was discussed. She objected, but was ruled out, and sat down. The first bye-law was then put to the meeting, and the sisters, &c., of the Bart.'s staff did vote, consequently, in my opinion, carried the rule. I understand it was because the question was asked by a provincial matron, during the discussion of the third bye-law, if sisters were to vote, Miss Stewart said no. Up to that time they had done so. I believe the first step was the great mistake of the meeting in the name of "Matrons' Council," which never ought to have been passed until the third bye-law was discussed. Now the Council is not a matrons' but a nursing council, and this is what has caused its unpopularity and prevented good and united work.

### THE HOSPITAL AT HAIFA.

Miss Harriet Smith writes: I have just returned from South Africa and am going out to the hospital at Haifa, Syria, to join Miss Allen, with whom I worked at Zanzibar. Bishop Blyth is starting this hospital and mission station in connection with the mission at Jerusalem, and Miss Allen writes that anything I can take out will be acceptable, and nothing can come amiss. I therefore trust that a ready response may be made to my appeal for surgical instruments, maternity appliances and waterproof sheets, also sheets, pillow-cases, blankets, carrying chair, bed tables, nurse's wallet, disinfectants, and clinical thermometers. For my own special work (maternity) I shall want everything. I see so many useful things noticed in The Hospital which really seem necessaries, but having been out of England so long, I feel ignorant of the best ways and means of getting help for the mission. Perhaps some of your readers may know of instruments, &c., lately the property of a doctor which the relatives would part with? I am starting for the Holy Land about October 1st, and shall be at Belford Lodge, Leamington Spa, until that date.

### ASSOCIATION OF ASYLUM ATTENDANTS.

"Certificated Attendant" writes: May I trespass on your valuable space with a few remarks anent the proposed Association of Asylum Attendants? Some few months ago a short paragraph appeared in The Hospital setting forth a proposal to form such an association, the objects of which should be to raise the status of asylum attendants and help them to secure better training; also to circulate amongst members a monthly journal to be called "Asylum News." I feel sure most experienced asylum workers will admit this is much needed, and could not be other than beneficial to all concerned. If there be any possible chance of the plan being brought to a successful issue I think it would be a pity to allow it to slide. I am convinced if taken up ably, with an energetic person at the head of affairs, much useful work might be done. It is much to be regretted that the majority of asylum attendants manifest so much indifference to their positions; if they could be induced to interest themselves more in the details of their work and endeavour to raise themselves and improve their positions, there would be some chance of obtaining the recognition and respect to which responsible work gives a title. I should be glad to hear of some steps being taken in connection with the above-mentioned association. If once established there is little doubt as to its ultimate success and beneficial effect on asylum workers in general.

⁂ We advise "Certificated Attendant" to write on this subject to Dr. Harding, Berrywood, Northampton.

### RED CROSS OF KIMBERLEY HOSPITAL.

Miss Annie Rose-Innes writes: In your issue of August 4th appear some remarks under the heading "Red Cross," which are calculated to prejudice nurses who go through their training and obtain certificates of competency as trained nurses in the Carnarvon Hospital at Kimberley, South Africa. I don't know what Colonial nurse could have been guilty of making such foolish remarks in reference to the "niceness and prettiness" of wearing a particular badge, but at the above hospital certain of the nurses who are adjudged worthy are invested with the Red Cross after a satisfactory term of service, and are entitled to wear that distinction. If it be not the custom in English hospitals to provide a decoration of competency, it cannot be objected that an important hospital in another part of the world may not mark its sense of the status of its trained nurses in any way it thinks best. To obtain the fullest information as to the Red Cross of Kimberley Hospital, I cannot do better than refer your correspondent to the Matron. I am sure she would at once give you its history and the details of investiture.

### KIMBERLEY HOSPITAL.

Miss Rose Aimée Blennerhassett, Lady Superintendent of the Civil Hospital, St. Helena, writes: By this mail I received The Hospital of June 2nd, which contains a letter from Dr. Draper Bishop about Kimberley Hospital. Having been Night Superintendent at this institution during the winter of 1890-91, I am well acquainted with the management of the hospital, and should like to say a few words in answer to Dr. Draper Bishop's letter. In the first place, to the best of my belief his statements are quite correct. The nurses at Kimberley are practically entirely in Sister Henrietta's hands; the "dress" is black and stuffy, the rule severe. But—before being engaged each nurse is supplied with a copy of the rules; she knows that her "dress" will be black and possibly stuffy; she knows that whilst at Kimberley she will never be allowed to go to a play or a concert, and that all amusement must be taken in the afternoon—never in the evening. If this seems hard, it must be borne in mind that many of the probationers at Kimberley are girls of 18 and 20, who are entrusted to Sister Henrietta's care by their parents just because the rule is strict, and a perhaps oppressively careful watch kept over the nurses. Also if Mr. Judge and the other members of the Hospital Board leave their nurses in the power of the Matron, it is only fair to explain who that Matron is. For something like twenty years Sister Henrietta has been associated with Kimberley. She nursed there in the tents and hovels of the rough mining camp, and under her auspices a great and efficient hospital has grown up. She has got together a staff of well-trained private nurses, organised an institution which can accommodate over 300 sick, and created a large training school for nurses. She has done this in the heart of Africa, and in the face of difficulties which must often have seemed insurmountable. Numbers of small hospitals scattered over South Africa are nursed and managed by Kimberley nurses. Sister Henrietta's private nurses not only care for the sick of Kimberley, but travel hundreds of miles by train and post-cart to lonely Veldt farms and outlying villages; whilst from Sister Henrietta's training school come the nurses who devote themselves to the lepers of Robbin Island. No doubt Kimberley Hospital is not a paradise; no doubt there are squabbles and discontents amongst the nurses. What institution is free from them? But the fact remains that few women have accomplished a nobler and more useful work than Sister Henrietta's, and the Kimberley Hospital Board may be excused for trusting a woman whose name is known and respected throughout the length and breadth of South Africa.

### The Book World for Women and Nurses.

[We invite Correspondence, Criticism, Enquiries, and Notes on Books likely to interest Women and Nurses. Address, Editor, The Hospital (Nurses' Book World), 428, Strand, W.C.]

Outlaw and Lawmaker. By Mrs. Campbell Praed. (Chatto and Windus. 3 vols.)

"Outlaw and Lawmaker" is a somewhat sensational title, nor do the three volumes of Mrs. Campbell Praed's latest work, on closer acquaintance, bely the startling promise of their name. Mrs. Campbell Praed has a talent for picturesque grouping that lifts her works above the level of the commonplace and makes them always, or nearly always, interesting reading; further, she has a rather convincing style. Not even the deft use of this last attribute, however, can make Morres Blake, ex-Fenian, outlaw and lawmaker of Leichardt's Land, anything but melodramatic and reminiscent of the "gentlemanly villain" of a bygone age. Elsie Valliant, the heroine, too, is not only very mercenary, but extremely foolish; but then the latter-day heroine is not required to be either attractive or comprehensible. Some of the incidents of the story are rather strained, notably the robbery of Lady Waveryng's jewels. At the first mention of the ill-fated diamonds it is impossible not to guess that they have been brought into the bush, against their owner's wishes, simply and solely for the purpose of allowing them to fall a prey to the enterprising bushranger, Moonlight. Nevertheless, in spite of faults, the book is eminently readable and interesting well-nigh from first to last, some of the minor characters—such as Lord Horace and his poor little wife, Ina—having a reality about them that is lacking in the principal figures. Some of the scenes are dramatic enough, notably that of Lord Horace's death, with the silly little woman with whom he had been flirting, sobbing, and confessing at his side, but on the whole the impression left by "Outlaw and Lawmaker" is that Mrs. Campbell Praed has done very much better things.

In Varying Moods. By Beatrice Harraden. (William Blackwood and Sons, Edinburgh and London.)

The talented authoress of this collection of short stories has already established for herself a considerable reputation as the writer of "Ships that Pass in the Night"; and those who have not already had an opportunity of reading several of these stories as originally published in *Blackwood's*, the *English Illustrated*, and other magazines have a real pleasure in store. The first and longest story in the series, namely, "At the Green Dragon," is perhaps the most masterly and impressive effort of the collection. It well deserves the foremost place in the volume, and puts the reader in a contented and indulgent mood for the perusal of what follows. There is much to remind one of the style of Olive Schreiner, and a higher compliment it is scarcely possible to pay. And there is something, too, suggestive of Stevenson as he reveals himself in "The New Arabian Nights," and this is more especially noticeable in "The Umbrella Mender" and in "The Clockmaker and His Wife," in both of which sketches the authoress is particularly happy, and combines a considerable amount of grim humour with much that is pathetic. The concluding sentences of the second of the above mentioned stories will justify our remarks as to the resemblance in style to Olive Schreiner. We quote them so that our readers may judge for themselves: "'When the fiddler sees the light he will come back,' they said to each other. They waited until the day broke, and the storm was hushed in sleep, and the fire died out. But the fiddler did not come back." Such is the leaven which leavens the whole book; and if the authoress has borrowed a few "literary tricks," we, as readers, ought to feel mightily thankful that she recognises what is worth borrowing. We do not for a moment contend that these stories shine only with a borrowed light. The reader will discover much that is original, and original in a peculiar line. We shall await further creations from the same pen with impatience.

A Cluster of Nuts. By Katherine Tynan. (Laurence and Bullen, 3s. 6d.)

A collection of slight sketches which first saw the light at different times and places must, almost of necessity, be unequal in merit; and it is no disparagement of Miss Tynan's delineation of Irish life to say that they differ considerably in quality. The pathetic element enters largely into the composition of them all, so much so, indeed, that occasionally just here and there it appears a little forced; an effect that is probably the result of a series of themes in the same key, and would not have been apparent had each been read as it was written separately. The authoress is most successful in a certain delicate handling of the slightest of incidents—little "snap-shots" of character rather than events. "Snap-shots," perhaps, is hardly the right word to apply; Miss Tynan's art resembles rather the loving idealisation of the painter than the crude realism of the Kodak. The story, or rather sketch—which many will think the best in the book—of the two poor emigrants and their old violin, is a perfect illustration of this delicacy of touch, and by itself is worth a wilderness of three-volume novels. Further, the Ireland of which Miss Tynan writes with the insight born of knowledge, is not the melodramatic Ireland with the accessories of brogue and boycott which is the stock background of a certain class of novelist; but a rustic, nonpolitical, poverty-stricken Ireland, or, saddest of all, an Ireland that her sons are sorrowfully driven to leave by the relentless pressure of their utmost need.

The Garden that I Love. By Alfred Austen. (1 Vol. London: Macmillan and Co.)

There is a quaintness and daintiness about Mr. Alfred Austen's artistic looking little volume that will probably gain for it many readers outside that fortunate section of the community who, like the author, have gardens which they own not merely by right of possession, but by right of the labour which is a labour of love. "The Garden that I Love" is simply the tale, or rather, description of a hobby—a description given with so light and loving a touch that something of the sweet greenness of a country lawn seems to hang about the book, read it where you will. Naturally the author exalts his own particular hobby above all others; but who shall find fault with him for that?—more especially when that hobby is one that, taken as a whole, brightens more lives than any other, and, as Mr. Austen sets forth, forms a bond of union between flower-lovers in any and every rank of life. The "human interest" of the book is decidedly small; the figures that appear in its pages here and there are curiously unreal, serving only to grace the lawns and orchards which Mr. Austen's soul loveth. In fact, the rather up to date Lamia seems at times a little out of place in the peaceful glamour of so ideal a garden, disturbing somewhat the half-lazy delight, which is the predominant feeling awakened by "The Garden that I Love."

### Books Received.

John Richardson and Co.

"Our State Hospitals." By Thomas M. Dolan, M.D.

Kegan Paul, Trench, Trubner and Co.

"Growing Children and Awkward Walking." By Thomas W. Munn, F.R.C.S.Eng.

**Periodicals and Pamphlets.**—London, Westminster Review, English Illustrated Magazine, Cornhill, Humanitarian, St. Nicholas, Therapeutic Gazette, Report on the Social Condition of the People, by J. Ryland, Verulam Review, Leisure Hour, Boys' Own Paper, Girls' Own Paper, Sunday at Home, Our Little Dots, Child's Companion, Light in the Home, Friendly Greetings, Cottager and Artisan, Report of Public Health and Sanitary Condition of the Parish of St. Mary, Battersea, Revue Générale des Sciences, Amateur Gardening, and Review of Reviews.

